# Isolated LOC in head trauma associated with significant injury on brain CT scan

**DOI:** 10.1186/s12245-017-0154-7

**Published:** 2017-09-25

**Authors:** Muhammad Waseem, Patrick Iyahen, Hilary Bryan Anderson, Kevin Kapoor, Ramnath Kapoor, Mark Leber

**Affiliations:** 1 Lincoln Medical Center, 234 East 149th Street, Bronx, NY USA; 2 St. George’s University, True Blue, Grenada; 3 Brooklyn Hospital Center, 121 Dekalb Ave, Brooklyn, NY 11201 USA

**Keywords:** Loss of consciousness, Traumatic brain injury, Glasgow Coma Scale (GCS), Brain CT scan

## Abstract

**Background:**

A report of loss of consciousness (LOC) is frequently considered reason enough to obtain a computed tomography (CT) scan in the evaluation of head trauma. We conducted this study to reduce exposure to radiation from CT, while still not overlooking clinically significant injuries.

**Objective:**

The objective of the study is to determine the correlation between LOC status and brain CT scan results in patients with blunt head trauma and to determine whether there is a subset of patients for whom CT scan need not be performed, without missing clinically significant intracranial injuries.

**Methods:**

This is a retrospective study conducted in the emergency department of an inner-city hospital. The patient population included patients ranging between 13 and 35 years of age, with blunt head trauma, who presented to the emergency department (ED) between January 2010 and December 2013. Patients were divided into two groups: “LOC” group and “no LOC” group. The results of brain CT scans from each group were compared with LOC status. For study purposes, “clinically significant” were those that required interventions or ICU hospitalization of at least 24 h or extended hospitalization. The results were analyzed using chi-square calculations.

**Results:**

During the study period, 494 patients were identified as having suffered head trauma. Of these, 185 (37.5%) reported LOC and 309 (62.5%) did not lose consciousness. In the LOC group, 15 (8.1%) had significant CT findings compared to 1.3% (4/309) of those without LOC (*p* < .001). Of the 4 who had no LOC and had significant brain CT findings, all 4 patients had positive physical findings of head, neck, or facial trauma. In the LOC group, only 1/15 (6.7%) had significant CT findings with a normal GCS of 15 and no physical signs of the head, neck, or facial trauma.

**Conclusions:**

A small proportion of patients with LOC had CT finding requiring intervention. Head trauma patients with no physical injuries to the head, neck, or face and a normal GCS had no significant brain CT findings. This raises the question of whether a routine brain CT scan should be obtained in patients with LOC, no physical findings, and a normal GCS after blunt head trauma.

## Background

Head trauma is a common reason for emergency department visits. Many patients who suffer blunt head trauma are at risk of traumatic brain injury (TBI). This affects approximately two million people in the USA each year [1]. In addition, there are 1.4 million annual hospitalizations for TBI [2]. A report of loss of consciousness is frequently considered an indication for obtaining a brain CT scan in the evaluation of patients with blunt head trauma [3–5]. In the USA, physicians routinely obtain CT scans for patients with abnormal Glasgow Coma Scale (GCS) scores and/or LOC. This study evaluated whether a report of LOC in a patient with blunt trauma, but an otherwise normal physical examination, warrants obtaining a brain CT scan.

## Methods

This is a retrospective study conducted in the ED of an urban hospital, which is a state-designated level I trauma center in New York City. It has a very busy emergency department with annual visits of approximately 120,000. This hospital has both emergency medicine and surgery residency training programs. The study population is comprised of individuals primarily from the South Bronx, between 13 and 35 years of age, with blunt head trauma, who presented to the ED between January 2010 and December 2013. CT scans were obtained at the discretion of the treating physicians.

### Inclusion criteria

For study purposes, we included only previously healthy patients between the ages of 13 and 35 years of age, who presented following blunt head trauma.

### Exclusion criteria

Patients with other medical conditions that may influence CT result, such as stroke, were excluded. Similarly, patients more than 35 years of age were excluded. Patients who were at higher risk for intracranial bleeding and those taking anticoagulant medications were also excluded. Patients who were suspected of substance abuse or intoxication were not included in this study.

### LOC definition

For study purposes, LOC was considered to be present if there was any reported period of unconsciousness following the traumatic event documented in the patient’s medical record.

Based on the LOC status, patients were divided into two groups: “LOC” group and “no LOC” group. The results of brain CT scans from each group were then compared with LOC status. In addition, we looked at GCS status of the patients exhibiting clinically important CT findings.

### Physical findings

For study purposes, physical findings were defined as injuries to the head, neck, and face or the presence of alterations in mental status.

### Outcome measure

“Clinically important” findings on CT scan either required surgical intervention or greater than 24-h ICU admission or increased length of hospitalization. These included intracranial hemorrhage (including subdural, epidural, or intra-parenchymal types), skull fracture. The presence of a chronic intracranial lesion or findings that did not require acute intervention or increased length of hospitalization were not considered clinically important in the acute management of trauma.

In addition, we collected the following data: patient age, presentation information, initial GCS score on arrival, mechanism of injury, and disposition status. All data collection, including a chart review of the patients in this study, were performed by the primary authors. All CT scans were interpreted by board certified attending radiologists. The SPSS 22.00 (IBM Corporation, New York) was used to analyze the data. A bivariate analysis was performed, and Pearson chi-square calculations was used.

#### Statement of declarations, consent, or ethics approval

This study received approval from the Institutional Review Board of the Lincoln Medical and Mental Health Center (IRB reference no. 13-023). Written informed consent was waived in retrospective studies of anonymized patient data under Ethical Guidelines for Medical and Health Research Involving Human Subjects.

## Results

For the study period, we reviewed data from 494 patients with blunt head trauma in whom brain CT scans were obtained. There were 361 (73.1%) males and 133 (26.9%) females. The median age was 24.0 years [IQR = 9 (75th–25th percentile)]. LOC was reported in 185 (37.5%) patients, whereas 309 (62.5%) patients did not lose consciousness.

### LOC group

Of the 185 patients who experienced LOC, 136 (73.5%) were male and 49 (26.5%) were female. LOC was self-reported in 54 (29.2%) and witnessed in 131 (70.8%) patients. Of all patients who sustained LOC, 15 (8.1%) patients had significant CT findings and 170 patients did not have any clinically significant finding on CT scan; odds ratio (OR) 6.73 (95% CI 2.2–20.6) *P* < .0001 (Table 1).

**Table 1:** LOC and brain CT result

	CT positive	CT negative
LOC	15	170
(*N* = 185)	(8.1%)	(91.9%)
No LOC	4	305
(*N* = 309)	(1.3%)	(98.7%)
Total	19	475
(*N* = 494)	(3.8%)	(96.2%)

Odds ratio (OR) 6.73 (95% CI 2.2–20.6) *P* < .0001 for LOC (no LOC/LOC) and CT scan positivity

### Non-LOC group

Of the 309 patients who did not report LOC, 225 (72.8%) were male and 84 (27.2%) were female. In this group, significant CT findings were identified in only four patients (1.3% = 4/309). CT was negative in 305 patients (98.7%). All 4 of the patients with significant CT findings had associated findings of trauma involving the head, neck, or face.

### Positive brain CT scan

Overall, 19 patients were identified with significant findings in their brain CT scans. Of these, 15/19 (78.9%) patients reported LOC and 4 were in the non-LOC group. The majority of these, 18/19 (94.7%), were male. Of these 15, LOC was reported by a witness in six patients and nine self-reported the LOC. Only 15 patients, 8.1% (15/185), in the LOC group had significant CT scan findings compared to 1.3% (4/309) in the non-LOC group (*p* < .0001). In the LOC group, only 1/15 (6.7%) had significant CT findings with a normal GCS of 15 and no physical signs of head, neck, or facial trauma.

Of the 4 in non-LOC group with significant CT finding, all 4 had physical signs of head, neck, or facial trauma.

### GCS score

Based on the Glasgow Coma Scale (GCS), we divided patients into two groups: GCS 15 and GCS < 15. In the GCS 15 group, 461 patients were identified. Of these, 449 (97.4%) were CT negative and 12 (2.6%) were CT positive (NPV 97.4%). In this sample, 33 patients had a GCS score less than 15. Of these, 7 (21.2%) had positive CT scans and 26 (78.8%) had negative CT scans (PPV 21.2%). The sensitivity of GCS < 15 in predicting positive CT scan was 36.8% while specificity was 94.5% [OR 10.1 (95% CI 3.7–27.7) *P* < .0001] (Table 2). However, when GCS < 15 and a positive LOC were combined, sensitivity reached 89.5% and NPV reached 99.3% [OR 13.7(95% CI 3.1–59.9) *P* < .0001 (Table 3).

**Table 2:** GCS status and brain CT result

	CT positive	CT negative
GCS 15	12	449
(*n* = 461)	(2.6%)	(97.4%)
GCS < 15	7	26
(*n* = 33)	(21.2%)	(78.8%)
Total	19	475
(*n* = 494)	(3.8%)	(96.2%)

Odds ratio (OR) 10.1(95% CI 3.7–27.7) *P* < .0001 of association between GCS less than 15 vs. GCS = 15 and CT scan positivity

**Table 3:** Combined GCS < 15 and LOC status and brain CT result

	CT positive	CT negative
GCS < 15 and/or LOC	17	182
(*n* = 199)	(8.5%)	(91.5%)
GCS 15 and/or no LOC	2	293
(*n* = 295)	(0.7%)	99.3%
Total	19	475
(*n* = 494)	(3.8%)	(96.2%)

Odds ratio (OR) 13.7(95% CI 3.1–59.9) *P* < .0001 of association between GCS less than 15 and/or LOC vs. GCS 15 and no LOC comparing CT positivity

Based on GCS, patients were further classified into minimal or normal (GCS 15), mild TBI (GCS 13–14), moderate TBI (GCS 9–12), and severe TBI (GCS 8 or below). We then compared GCS score with the CT scan interpretation. The results were as follows: minimal or normal 461 (93.32%), mild TBI 17 (3.44%), moderate TBI 4 (0.81%), and severe TBI 12 (2.43%). In the minimal or normal group, CT scans were normal in 452 (98%) patients, whereas 9 (2%) showed clinically important CT findings. In the moderate TBI group, 2 CT scan (50%) were normal and 2 (50%) were positive. In the severe TBI group, 6 (50%) CT scan were negative and 6 (50%) showed positive findings; *P* < .001 (Table 4).

**Table 4:** GCS score and CT scan result

GCS score	CT scan result	
	Negative	Positive
Minimal or normal (GSCS 15)	452 (98%)	9 (2%)
*n* = 461 (93.32%)		
Mild GCS (13–14)	15 (88.2%)	2 (11.8%)
*n* = 17 (3.44%)		
Moderate (GCS 9–12)	2 (50%)	2 (50%)
*n* = 4 (0.81%)		
Severe (GCS 8 or below)	6 (50%)	6 (50%)
*n* = 12 (2.43%)		

*P* < .001 between mild to severe head injury and rate of CT scan positivity

Table 5 lists the characteristics of patients in this study with positive brain CT scans. There were three trauma causes or mechanisms identified in patients with positive CT scans: assault, motor vehicle crash (MVC), and falls. Of the 19 patients with significant CT findings (CT+), six were assaulted; two had falls subsequent to the assault; and eight were injured in motor vehicle crashes (MVC). The primary mechanism was a fall in five patients.

**Table 5:** Patient characteristics with positive brain CT scan result

Age (years)	Gender	Mechanism	GCS	CT result
LOC positive (*n* = 15)				
17	Male	MVC	15	Subdural hemorrhage
19	Male	Fall	15	Epidural hemorrhage/fracture
				Midline shift
20	Male	MVC	10	Intracranial hemorrhage
22	Male	MVC	6	Depressed skull fracture
				Epidural/intra-parenchymal hemorrhage
22	Male	Fall	12	Depressed skull fracture
				Subarachnoid hemorrhage
25	Male	Fall/assault	15	Skull fracture
25	Male	MVC	6	Skull fracture
26	Male	Assault	15	Subarachnoid hemorrhage
28	Male	Assault	15	Depressed skull fracture
				Pneumocephalus
29	Male	Assault	7	Epidural hemorrhage
34	Male	MVC	14	Subdural hemorrhage
35	Male	Assault	15	Skull fracture
24	Male	Fall	3	Severe multiple fractures
				Intra-parenchymal hemorrhage
22	Male	MVC	6	Depressed skull fracture
				Intra-parenchymal/epidural hemorrhage/midline shift
22	Female	MVC	3	Acute axonal injury
LOC negative (*n* = 4)				
18	Male	Fall	13	Skull fracture
19	Male	Fall	15	Subdural hemorrhage
21	Male	Assault	15	Depressed skull fracture
30	Male	Fall	15	Skull fracture

We categorized the patients into pediatric and adult groups. There were 57 (11.5%) pediatric patients under the age of 19 years and 437 (88.5%) adults. In adults, 164 [164/437 (37.5%)] reported LOC, whereas in 21 pediatric patients, LOC was present [21/57 (36.8%)]. In terms of CT scan results, one pediatric patient had a positive CT scan while 18 adults had a positive CT scan with clinically important injuries [18/437 (4.1%) *p* = 0.38.

## Discussion

Loss of consciousness is common in patients with blunt head trauma and is an important factor influencing the decision to order a CT in the ED. In a large cohort study of mild TBI patients, CT scan was obtained in 91% [6].

Many physicians have questioned the relationship between a history of LOC and TBI [7, 8]. Despite this, the practice of obtaining CT scans in patients with LOC has remained unchanged, and CT has been a mainstay of diagnostic testing in the ED. A brain CT scan is the test most commonly ordered in the evaluation of patients with head trauma [9, 10]. Each year, more than 1 million head CTs are obtained for head trauma [11, 12]. A report has revealed that the rate of head CT use has increased 35.7% (range = 34.5 to 46.8%) for all ages between the years 2006 and 2011 [13].

This study revisited the question of whether a brain CT scan is always required in patients with blunt head trauma who report LOC on arrival in the ED. Using only clinical evaluation certainly reduces CT scan use, but may not eliminate the diagnosis of intracranial injury. For many physicians, the idea of missing the diagnosis of any intracranial injury is unacceptable. It is possible that the concern of litigation may play a role in medical decision-making, and some would not accept missing the diagnosis of any intracranial injury regardless of its significance. A New Orleans Criteria study has suggested that CT use could be avoided in patients with minor head injury in the absence of any of the following: headache, emesis, age > 60 years, drug or alcohol intoxication, posttraumatic seizure, physical evidence of trauma above the clavicle, and short-term memory deficits [14]. These “New Orleans Criteria” were created to capture all intracranial injuries, including those which did not require neurosurgical intervention. To improve specifically detecting clinically important brain injury, the Canadian CT Head Rule allowed a patient with GCS 13-15 within 2 h of the injury to forego a head CT, but included other criteria such as age < 16 and mechanism of injury that would automatically require a brain CT scan. To identify patients with any clinically important brain injury, the Canadian CT Head Rule also included amnesia as one of the criteria [15].

In our study, LOC was reported in 37.5% of patients who presented with blunt head trauma. Fifteen of these patients (8.1%) had clinically important findings on CT scan. If no physical findings were found in the head, neck, or face, and there was no alteration of consciousness, only one patient had a significant CT scan finding.

In this sample, the majority of patients presenting following blunt head trauma were male (73.1%) and 18 out of 19 (94.7%) of the CT positive patients were also in male patients. This correlates well with the general trend for males being at a higher risk for involvement in MVC and trauma. In order to minimize head injuries, perhaps primary prevention strategies should be directed towards the male population.

We also reviewed the association of GCS and CT scan results. In this study, 7 patients (21.2%) with GCS < 15 had a positive CT scan GCS (*n* = 33). In a previous study, patients with GCS of 13 had a five times greater risk of having intracranial injury [16]. Another study suggested receiver operating characteristic areas greater than 0.65 with LOC if the GCS score was less than 15 (0.692) [17].

This study had several potential limitations. First, the retrospective design of this study meant that determination of LOC status was dependent on the documentation in the medical record. If it was not recorded, it was assumed that there was no LOC. Second, the study was underpowered to detect differences in physical findings. We acknowledge that sometimes, an accurate report of LOC may be difficult to ascertain because of the lack of a witness to the traumatic event. In addition, there may have been difficulties in determining whether the patient had actually been unconscious. We also do not know the reliability of the LOC reported by the patient or witness in the field. It is possible that the patients who reported no LOC could have thought they were lucid during the trauma, but may have briefly lost consciousness and could not remember the entire event.

## Conclusions

In this limited sample, there was only one patient with a significant CT scan finding with isolated LOC, GCS of 15, and otherwise no physical injuries. The practice of routinely ordering brain CT scans should be reconsidered. Until more definite evidence becomes available however, judgment should be used when evaluating patients with blunt head trauma with associated LOC, when deciding whether to obtain a brain CT scan.
